# Directions for Work in the Histological Laboratory, More Especially Arranged for the Use of Classes in the University of Michigan

**Published:** 1896-05

**Authors:** 


DIRECTIONS FOR WORK IN THE HISTOLOGICAL LABORATORY, more especially arranged for the use of classes in the University of Michigan. By G. CARL HUBER, M. D., Assistant Professor of Histology and Embryology. Second edition. Geo. Wahr, Publisher, Ann Arbor, Mich. 1895.

As indicated, this very practical little work was intended for use in the working laboratory and is arranged in the form of lessons, each lesson taking up several types of tissues, with a description of the hardening process, staining and mounting media employed. The tissue is carefully described histologically and the student required to study the various characteristics of the tissue under different powers and to make drawings of the same on the blank pages inserted after each lesson. Professor Huber's plan is most ingenious and deserves to be imitated by the histologists and pathologists of all our medical schools. His labors deserve a greater field of appreciation than in his own school, and this excellent but modest little work could well be adopted, and with profit, by every medical college in America. The publisher has aided materially in making the book so pleasing and acceptable and deserves great credit. W. C. K.

